# Validation of a Semi-Quantitative Food Frequency Questionnaire for Argentinean Adults

**DOI:** 10.1371/journal.pone.0037958

**Published:** 2012-05-25

**Authors:** Mahshid Dehghan, Silvia del Cerro, Xiaohe Zhang, Jose Maini Cuneo, Bruno Linetzky, Rafael Diaz, Anwar T. Merchant

**Affiliations:** 1 Population Health Research Institute, McMaster University, Hamilton, Ontario, Canada; 2 Clinical Research Department, Estudios Clínicos Latinoamérica (ECLA), Rosario, Argentina; 3 Department of Epidemiology and Biostatistics, Arnold School of Public Health, University of South Carolina, Columbia, South Carolina, United States of America; University of Cordoba, Spain

## Abstract

**Background:**

The Food Frequency Questionnaire (FFQ) is the most commonly used method for ranking individuals based on long term food intake in large epidemiological studies. The validation of an FFQ for specific populations is essential as food consumption is culture dependent. The aim of this study was to develop a Semi-quantitative Food Frequency Questionnaire (SFFQ) and evaluate its validity and reproducibility in estimating nutrient intake in urban and rural areas of Argentina.

**Methods/Principal Findings:**

Overall, 256 participants in the Argentinean arm of the ongoing Prospective Urban and Rural Epidemiological study (PURE) were enrolled for development and validation of the SFFQ. One hundred individuals participated in the SFFQ development. The other 156 individuals completed the SFFQs on two occasions, four 24-hour Dietary Recalls (24DRs) in urban, and three 24DRs in rural areas during a one-year period. Correlation coefficients (r) and de-attenuated correlation coefficients between 24DRs and SFFQ were calculated for macro and micro-nutrients. The level of agreement between the two methods was evaluated using classification into same and extreme quartiles and the Bland-Altman method. The reproducibility of the SFFQ was assessed by Pearson correlation coefficients and Intra-class Correlation Coefficients (ICC). The SFFQ consists of 96 food items. In both urban and rural settings de-attenuated correlations exceeded 0.4 for most of the nutrients. The classification into the same and adjacent quartiles was more than 70% for urban and 60% for rural settings. The Pearson correlation between two SFFQs varied from 0.30–0.56 and 0.32–0.60 in urban and rural settings, respectively.

**Conclusion:**

Our results showed that this SFFQ had moderate relative validity and reproducibility for macro and micronutrients in relation to the comparison method and can be used to rank individuals based on habitual nutrient intake.

## Introduction

Epidemiological studies have indicated strong associations between habitual dietary intake and chronic diseases such as cardiovascular disease, obesity and cancer 1–3. To understand the association between diet as a modifiable risk factor and chronic diseases, a measure of individuals’ long term dietary intake is needed. Habitual dietary intake can be evaluated by different dietary methods including food records, multiple 24-hour Dietary Recalls (24DRs), and Food Frequency Questionnaires (FFQ). Food record has been shown to be an accurate method for measuring individuals’ long-term intake but it requires participant motivation and literacy. Similarly, 24DRs rely on the respondents’ motivation, awareness of their food intake and literacy. Since the conceptual exposure in studies of chronic disease is long term diet, the FFQ is suitable for measuring this exposure as the FFQ usually assesses the habitual dietary intake over one year. The FFQ is not the method of choice for measuring individual’s absolute intake and the most common use of FFQs is to rank individuals by their food and nutrient intakes [4]. Since feasibility**,** cost and time are important limiting factors in large epidemiological studies, an FFQ is the best method of choice for assessing participants long term food intake. However, an FFQ developed for one population cannot be readily used in another population because different groups of people eat different foods and incorrect estimations of exposure may lead to false associations between dietary exposure and health outcome [5].

We developed and validated a SFFQ to be used for assessing the dietary intake of Argentinean adults participating in an ongoing cohort study called Prospective Urban and Rural Epidemiological study (PURE). The method of SFFQ development and validation is carefully standardized for all PURE participating countries [6]–[10]. The aim of this study was to evaluate validity and reproducibility of the constructed SFFQ in assessing intake of some nutrients of interest in both urban and rural areas of Argentina. As far as we are aware, only one other validation study was conducted in an urban area of Argentina [11]. The present study enrolled individuals from both urban and rural areas.

## Methods

The PURE study is a large ongoing prospective cohort being conducted in seventeen low, middle and high income countries and has recruited approximately 153,996 men and women, aged 35 to 70 years. The main objective of the PURE study is to examine the association between societal influences on human lifestyle, and risk factors of non-communicable diseases. The design and main findings of the PURE study have previously been reported [12], [13].

### SFFQ Development

We collected a 24-hour dietary recall (24DR) from 100 participants residing in urban and rural areas of Rosario in Santa Fe province. The most commonly reported food items were compiled as a food list and pre-defined portion size were assigned for each food item. To ensure face and content validity of the SFFQ, two expert nutritionists (MD and S del C) checked the food list and if nutrient rich or discriminating foods were missing, those foods were added to the list and structured the food list as a SFFQ. The SFFQ consisted of a food list, pre-determined portion size, and frequency of intake. Standard portions sizes (such as a glass of milk, one medium apple or a cup of ice-cream) that had been reported in 24DRs were assigned for each food item. Foods were classified into the following categories: milk and dairy products, fruits, vegetables, meat, cereals, soups, beverages, sweet and baked goods and nuts. The intake frequencies consisted of nine categories ranging from never to more than 6 times/day. Frequencies were formatted to recall food consumption during the last year.

To standardize the method of data collection and reduce inter-interviewers error, we prepared a detailed protocol for 24DR administration. The protocol consisted of two parts: a brief theoretical overview of SFFQ development and validation, and a step-by-step practical overview of the process to be followed in administration of the 24DR. The senior nutritionist in Argentina (S del C) conducted the training session for all interviewers. During the training session, it was emphasized that we were interested in everything that participants had eaten and drank during the previous day and the respective amounts. Interviewers were asked to avoid non-verbal cues indicating surprise or disapproval at the subject’s eating patterns, and the concept of “social desirability” was extensively discussed.

### Development of Food Photograph Atlas for Mixed Dishes

To reduce the bias report related to the amount of food consumption, we constructed food photographs to assist respondents in the estimation of portion sizes. For each mixed dish we created eight portion sizes and took a picture from each one. We assumed the average portion size based on the most frequently reported portion size in the administered 24DRs during SFFQ development, or based on the one that was chosen by the most popular cook book. The weight interval between serving sizes in each series of photographs corresponded to a fixed “increment” equal to one-fourth of the usual portion size for each food. For example, if the average portion size for rice was 280 g, we divided 280 by 4 and each increment was 70 g.

### SFFQ Validation

**Figure 1:**
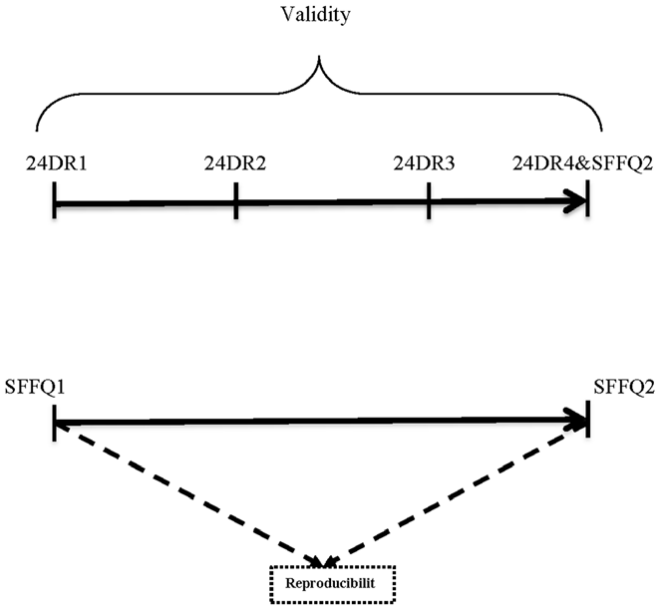
The design of SFFQ validation in urban setting.

We chose 24DR as the comparison method for both urban and rural setting. In rural area we collected four 24DRs over the period of one year. However, participants in rural area only agreed to three days of 24DRs. Hence, the number of days varied between urban and rural areas for the comparison method. For both settings, one of the 24DRs was administered during the weekend. The first 24DR was completed with the first SFFQ (SFFQ1) and the last 24DR administered with the second SFFQ (SFFQ2). The design of the study is shown in Figure 1. During 24DR administration, we used our food atlas for mixed dishes and “A photographic Atlas of Food Portion Size” [14], for single food items such as milk or cheese, to assist participants in visualizing portion sizes. To test reproducibility, the SFFQ was administered twice approximately one year apart.

### Food Composition Database

To estimate daily intake of energy, macro and micro-nutrients, a food composition table was required, and as the tool is to be used for an international study, a food composition database containing nutrient estimates was developed allowing comparisons between PURE countries [10]. The nutrient database was primarily based on the United States Department of Agriculture food composition database and was modified appropriately with reference to Argentina food composition tables. Based on the food’s nutrient profile, the daily nutrient intake for each individual was calculated.

Information regarding demographic characteristics (age, sex, education, etc.) was obtained at the first visit. Trained research assistants who were trained for the PURE study measured the weight and height of participants. Body weight was measured with a digital scale to the nearest 100 g while participants wore no shoes and only light clothing; height was measured to the nearest 1 cm. To reduce missing values and measurement error, 24DRs and SFFQ were administered by trained interviewers.

### Ethics Statement

The study received approval from Hamilton Health Sciences/McMaster Health Sciences Research Ethics board and human research protection (OHRP) registration in Argentina. Written informed consent was obtained from all participants in the study.

### Analysis

We computed the mean and standard deviation for each nutrient obtained from SFFQ1, SFFQ2, and 24DRs for urban and rural areas separately. We log transformed the data to improve the normality distribution. Validity of the SFFQ was compared with the average of multiple 24DRs using Pearson correlation coefficients. The correlation coefficients with adjustment for total energy were computed by the residual model [4]; however, energy adjustments did not improve the correlations. Also, de-attenuated correlations were calculated to remove the within-person variability. Relative agreement between the two methods was tested by cross-classification of the nutrient score and estimation of the proportion of subjects who were classified by the two methods into same, adjacent, and extreme quartile. To assess the “limits of agreement” between SFFQ2 and 24DRs, the Bland-Altman method [15] was performed for energy, protein, fat, and carbohydrate intake. The differences of mean between the two methods were plotted against the average of the two methods for each macro-nutrient. Pearson correlation and Intra-class Correlation Coefficient (ICC) were used to estimate the reproducibility of the SFFQ. All analyses were conducted separately for urban and rural areas. All statistical analyses were performed using SAS version 9.1 (SAS Institute Inc. SAS/STAT) and STAT version 10.0.

## Results

Overall, 170 women and men participated in the validation study; however, 14 people were removed from the present analysis as their estimated daily energy intake was less than 500 kcal/d or >4500 kcal/d [16] or did not have two administered SFFQs or required number of 24DRs. The results presented here are based on the dietary assessment of 116 women and 40 men aged 52.7 (9.5) y who completed two SFFQs and the required number of 24DRs.

The demographic characteristics of participants are shown in **Table 1**. Participants in the urban area were slightly younger, more educated, and had a lower BMI than their rural counterparts. We compared the demographic characteristics of those participants who did not complete four 24DRs (urban) or three 24DRs (rural), and two SFFQs with other participants. The mean age, BMI, and level of education was similar between the two groups. In urban setting, the mean daily energy and some nutrient estimates by an average of 24DRs, SFFQ1 and SFFQ2 were similar, but FFQ2 over-estimated intake of some nutrients (such as carbohydrate, calcium and phosphorous) **(** **Table 2** **)**. In rural area, SFFQ2 underestimated the participants’ dietary intake in comparison with the average of 24DRs for 10 out of 18 nutrients.

**Table 1:** Demographic characteristics of individuals who participated in validation study, urban (n = 89) and rural (n = 67) areas.

	Overall	Urban		Rural	Excluded
Age mean (sd)	52.7 (9.5)		52.2 (9.1)	53.3 (9.9)	52.6 (10.9)
BMI mean (sd)	30.6 (6.6)		30.2 (5.9)	31.2 (7.4)	32.0 (7.5)
Marital status %					
Never married	10.9	11.2		10.5	0
Currently married	64.7	58.4		73.1	76.9
Common law	6.4	10.1		1.5	7.7
Widowed	7.7	6.7		9.0	15.4
Separated/divorced	10.3	13.5		6.0	0
Education %					
None	11.5	9.0		14.9	28.6
Primary	64.1	62.9		65.7	64.3
High School	19.2	24.7		11.9	7.1
College/University	5.1	3.4		7.5	0

**Table 2:** Mean (sd) daily nutrient intake estimated by the average of 24DRs and two SFFQs in urban (n = 89) and rural (n = 67) areas.

	Urban			Rural		
	24DR	SFFQ1	SFFQ2	24DR	SFFQ1	SFFQ2
Energy/Kcal	2270.0 (822.0)	2928.0 (1120.0)	2267.0 (848.0)	2340.0 (753.0)	2265.0 (886.0)	2097.0 (665.0)
Protein/g	93.0 (42.4)	115.4 (34.8)	92.9 (29.3)	98.0 (39.22)	88.4 (33.2)	80.0 (20.7)
Fat/g	93.0 (37.7)	115.7 (54.6)	82.0 (40.4)	106.3 (40.0)	89.3 (48.6)	74.8 (29.5)
Carbohydrate/g	268.2 (100.0)	353.3 (146.4)	288.5 (111.1)	248.7 (86.4)	266.7 (106.2)	268.8 (93.4)
Fiber/g	23.6 (10.2)	25.4 (10.1)	22.7 (9.1)	20.2 (7.3)	19.8 (11.6)	20.5 (6.8)
Calcium/mg	675.0 (280.7)	1184.0 (407.0)	954.6 (351.8)	689.4 (323.2)	890.6 (495.6)	818.0 (374.9)
Iron/mg	15.4 (5.7)	18.8 (6.4)	15.2 (5.1)	16.1 (5.9)	14.1 (5.0)	13.8 (4.2)
Phosphorus/mg	1190.0 (440.0)	1821.0 (544.0)	1431.0 (484.5)	1144.0 (403.0)	1333.0 (552.0)	1226.2 (415.7)
Potassium/mg	2386.0 (867.0)	3816.0 (1296.0)	3309.0 (1169.0)	2285.0 (749.0)	3088.0 (1421.5)	2850.0 (911.9)
Sodium/mg	3159.0 (1301.0)	4474.5 (1584.0)	3582.0 (1223.7)	3373.0 (1221.0)	3257.0 (1212.0)	3269.5 (1052.3)
Vitamin C/mg	86.2 (65.9)	162.8 (107.0)	140.9 (84.8)	63.7 (49.8)	136.3 (114.5)	129.8 (63.3)
Thiamin/mg	1.7 (0.6)	2.1 (0.7)	1.7 (0.6)	1.8 (0.7)	1.6 (0.6)	1.7 (0.6)
Riboflavin/mg	1.8 (0.7)	2.7 (0.8)	2.2 (0.8)	1.9 (0.8)	2.1 (0.9)	2.0 (0.6)
Folate/mg	330.5 (127.9)	498.4 (176.0)	417.6 (134.4)	371.4 (157.9)	368.4 (141.7)	395.53 (121.2)
Retinol/mg	254.2 (323.6)	388.0 (211.4)	284.9 (177.6)	315.9 (771.6)	367.4 (290.5)	309.7 (211.5)
SFA/mg	33.3 (15.5)	43.3 (21.3)	30.8 (16.7)	33.0 (14.8)	32.9 (17.2)	26.4 (11.8)
PUFA/mg	14.1 (5.2)	15.9 (7.7)	11.1 (5.3)	22.4 (7.8)	13.5 (13.7)	12.2 (6.5)
Cholesterol/mg	321.0 (163.9)	533.4 (281.0)	404.1 (198.9 )	330.9 (180.0)	387.1 (184.2)	318.9 (108.3)

**Table 3** posits the correlation coefficients between SFFQ2 and mean of 24DRs. For both urban and rural settings energy adjustment did not improve the correlation (data not shown). For urban participants crude correlation coefficients between SFFQ2 and 24DRs varied from 0.2 (retinol) to 0.47 (carbohydrate) and de-attenuation improved the correlation coefficients for all nutrients, the highest correlation coefficient was found for iron (0.62) and the lowest for retinol (0.30). In rural setting, the de-attenuated correlations for all nutrients were greater than 0.46 except for vitamin C (0.35) and retinol (0.41). We observed an unpredictable de-attenuated correlation for total fat, potassium, Poly Unsaturated Fatty Acids (PUFA), and saturated fatty acids (data not shown).

**Table 3:** Validity Pearson correlation coefficient between daily consumption of nutrients estimated by SFFQ2 vs. 24DRs in urban (n = 89) and rural (n = 67) area.

	Urban		Rural	
	r	r De-attenuated	r	r De-attenuated
Energy/Kcal	0.44	0.51	0.35	0.53
Protein/g	0.26	0.39	0.33	0.53
Fat/g	0.38	0.50	0.34	–
Carbohydrate/g	0.47	0.57	0.33	0.46
Fiber/g	0.28	0.33	0.46	0.65
Calcium/mg	0.35	0.49	0.46	0.68
Iron/mg	0.36	0.62	0.37	0.63
Phosphorus/mg	0.32	0.48	0.33	0.75
Potassium/mg	0.37	0.50	0.40	–
Sodium/mg	0.32	0.51	0.40	0.90
Vitamin C/mg	0.31	0.41	0.26	0.35
Thiamin/mg	0.37	0.47	0.37	0.63
Riboflavin/mg	0.36	0.46	0.34	0.56
Folate/mg	0.39	0.53	0.47	0.71
Retinol/mg	0.20	0.30	0.20	0.41
SFA/mg	0.40	0.52	0.40	–
PUFA/mg	0.25	0.48	0.11	–
Cholesterol/mg	0.30	0.50	0.30	0.57

**Figure 2:**
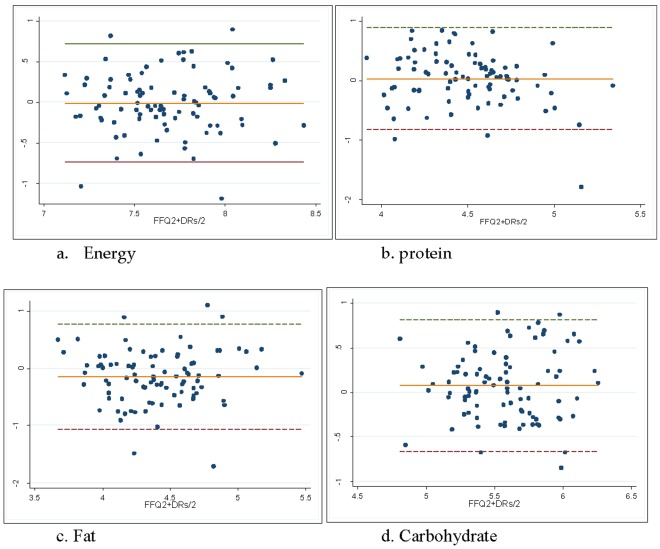
Bland and Altman plots for macronutrients with the mean difference and limits of agreements. Bland and Altman plots for a. energy, b. protein, c. fat, and d. carbohydrate with the pone.0037958.g003.tifmean difference (solid line) and 95% limits of agreements (Dashed lines) in urban areas.

The cross classification of daily nutrient intakes measured by SFFQ2 and 24DRs are shown in **Table 4**. In urban area, for most of the nutrients, the proportion of subjects classified into the exact same quartile varied from 24.7% (fibre) to 41.6% (energy) and the mean of disagreement between the two methods (extreme quartile) was 6.1%. In rural setting the exact agreement varied from 16.4% (for phosphorous and PUFA) to 49.3% (for fibre) and on average only 5.8% of participants classified into extreme quartile (disagreement) (**Table 4**). To illustrate the limits of agreement between two methods, we plotted the Bland-Altman scatter plots for daily energy, protein, fat and carbohydrate intakes for urban and rural settings (**shown in** **Figures 2** **and** **3**). In urban area, the mean difference for energy was small and indicated that SFFQ slightly (1%) underestimated daily energy intake (0.99, 95% CI; 0.48–2.06), but the underestimation was higher (10%) in rural area (0.90, 95% CI; 0.43–1.89). For energy and all macro-nutrients, a few numbers of individuals fell outside the limit of agreements and for all measurements the mean differences were not associated with the means of the two methods, confirming an acceptable level of agreement between the two methods.

**Table 4:** Cross classification of intake of nutrients and mean of four 24DRs SFFQ2 vs. DRs in urban (n = 89) and rural (n = 67) area.

Nutrient	Urban				Rural			
	Same quartile	Adjacent quartile	One quartile apart	Opposite quartile	Same quartile	Adjacent quartile	One quartile apart	Opposite quartile
Energy/Kcal	46.1	33.7	15.7	4.5	26.9	38.8	28.4	6.0
Protein/g	34.8	46.1	11.2	7.9	31.3	43.3	17.9	7.5
Fat/g	32.6	42.7	20.2	4.5	28.4	41.8	20.9	9.0
Carbohydrate/g	37.1	43.8	15.7	3.4	25.4	43.3	26.9	4.5
Fiber/g	24.7	43.8	25.8	5.6	49.3	32.8	14.9	3.0
Calcium/mg	31.5	46.1	16.9	5.6	34.3	44.8	14.9	6.0
Iron/mg	34.8	37.1	20.2	7.9	26.9	49.3	19.4	4.5
Phosphorus/mg	36.0	34.8	23.6	5.6	16.4	46.3	32.8	4.5
Potassium/mg	33.7	40.4	21.3	4.5	37.3	41.8	14.9	6.0
Sodium/mg	29.2	42.7	19.1	9.0	31.3	38.8	26.9	3.0
Vitamin C/mg	33.7	38.2	19.1	9.0	32.8	37.3	22.4	7.5
Thiamin/mg	34.8	33.7	24.7	6.7	31.3	46.3	17.9	4.5
Riboflavin/mg	32.6	41.6	22.5	3.4	32.8	40.3	19.4	7.5
Folate/mg	30.3	41.6	24.7	3.4	37.3	43.3	17.9	1.5
Retinol/mg	27.0	42.7	19.1	11.2	29.9	47.8	16.4	6.0
SFA/mg	34.8	42.7	18.0	4.5	35.8	40.3	19.4	4.5
PUFA/mg	36.0	32.6	25.8	5.6	16.4	38.8	35.8	9.0
Cholesterol/mg	33.7	43.8	14.6	7.9	35.8	37.3	16.4	10.4

**Figure 3:**
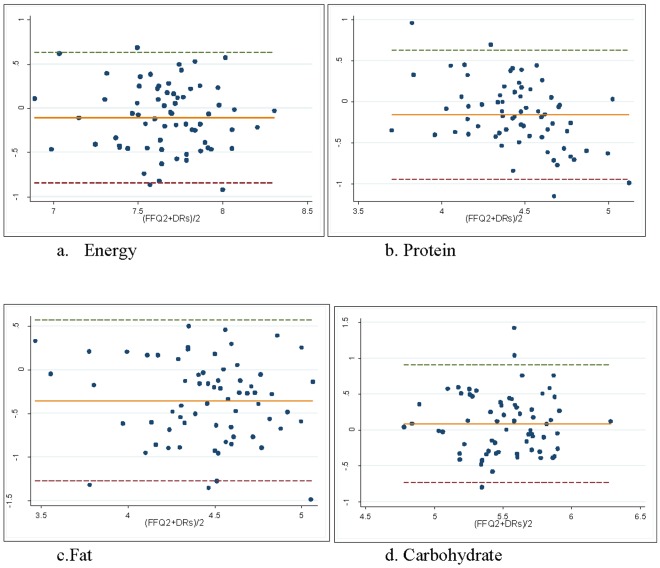
Bland and Altman plots for macronutrients with the mean difference and limits of agreements in rural. Bland and Altman plots for a. energy, b. protein, c. fat, and d. carbohydrate with the mean difference (solid line) and 95% limits of agreements (Dashed lines) in rural areas.

The results of repeatability between two SFFQs are shown in **Table 5**. The Pearson correlations (unadjusted) between nutrient intakes assessed by two SFFQs varied from 0.3–0.56 in urban and 0.32–0.60 in rural setting. For urban area, we found the lowest ICC for phosphorus (0.10) and the highest ICC for vitamin C (0.54), and for rural setting, the ICCs between two FFQs tended to be higher than urban and varied from 0.33 to 0.60.

**Table 5:** Repeatability, Pearson correlation coefficient between two SFFQs in urban (n = 89) and rural (n = 67) areas.

Nutrient	Urban		Rural	
	r	ICC	r	ICC
Energy/Kcal	0.50	0.32	0.44	0.43
Protein/g	0.31	0.16	0.41	0.40
Fat/g	0.50	0.25	0.52	0.50
Carbohydrate/g	0.50	0.40	0.33	0.33
Fiber/g	0.47	0.44	0.54	0.47
Calcium/mg	0.30	0.20	0.35	0.34
Iron/mg	0.44	0.32	0.42	0.43
Phosphorus/mg	0.30	0.10	0.40	0.40
Potassium/mg	0.47	0.41	0.51	0.50
Sodium/mg	0.45	0.31	0.32	0.33
Vitamin C/mg	0.56	0.54	0.40	0.37
Thiamin/mg	0.40	0.30	0.36	0.35
Riboflavin/mg	0.31	0.20	0.37	0.36
Folate/mg	0.41	0.33	0.35	0.33
Retinol/mg	0.54	0.41	0.60	0.60
SFA/mg	0.50	0.30	0.50	0.42
PUFA/mg	0.36	0.20	0.51	0.50
Cholesterol/mg	0.46	0.32	0.44	0.40

## Discussion

We developed and evaluated the validity and reliability of a SFFQ in urban and rural areas of Argentina. Our results indicated that the SFFQ had moderate to good relative validity (varied from 0.3 to 0.90) and moderate reproducibility (varied from 0.3 to 0.6) for most macro and micro-nutrients. We observed high agreement between the two methods in quartile categorization and more than 60% of participants in rural and 70% of individuals in urban were correctly classified into the same or adjacent quartiles. The Bland-Altman plots depicted the acceptable level of agreement between the two methods.

In the present study, 156 women and men were enrolled, which was similar to a large number of the previous studies [17]–[20]. However, different sample sizes of 44 [21] to more than 850 [22] have been reported, but the number of participants for most studies has been less than 200 individuals [23]. Also, for assessing the absolute agreement between FFQ and 24DRs using Bland-Altman, at least 50 and preferably 100 participants are required. Our sample size also met the requirements of the Bland-Altman method.

An FFQ consists of a food list and the number of food items included in FFQ varies from 5 to 350 items. The number of food items in the food list depends on the objective of the study, food availability, and variability of food consumption in the population under study. The number of food items in our SFFQ (96 food items) is considered to be a reasonable number [5], especially for rural setting with ethnically homogenous population and low foods variability.

The association between FFQ and comparison method is usually assessed by correlation coefficients [4], and due to various measurement errors for each dietary assessment method, the observed correlation coefficients are a measure of relative validity. In urban setting, we found moderate to high crude correlations between SFFQ2 and 24DRs, and similar correlation coefficients have been reported by previous studies [21], [24]. In rural area, for most nutrients, the crude correlations between 24DRs and SFFQ2 were moderate. Comparing crude correlation coefficients in urban and rural setting, for some nutrients (such as protein, fibre and, calcium) we observed higher correlations between SFFQ2 and 24DRs in rural than urban areas. The higher correlation may have resulted from a homogenous diet in rural settings. For example, the main sources of fibre are fruits and vegetables and in rural areas of developing countries foods such as vegetables and fruits are cash crops, limiting their availability and affordability for rural residence.

For both urban and rural areas, energy adjustment did not improve the observed correlations for some nutrients. This may indicate that the SFFQ to some extent systematically over/under estimated intake of those nutrients. However, error in over/under estimation by SFFQ is expected. Likewise, Xia et al. and Jackson et al. reported that energy adjustment did not improve the observed correlations in their studies [20], [24]. To correct for day to day variation in food intake, the de-attenuated correlations were computed. Due to correction for within person variation, de-attenuated correlations are usually higher than crude correlations. Our findings in both urban and rural areas showed that for more than 80% of nutrients, de-attenuation improved the correlations and they were greater than >0.4, which is considered as valid. However, in rural settings, we observed unpredictable de-attenuated correlations for total fat, potassium, PUFA, and saturated fatty acids. Similar to our findings, Segovia-Siapco et al. [25] found very high correction factors for 13 out of 32 nutrients. The high correction factor may have been observed because of our small sample size (n = 67), and increased within person variation in intake of those nutrients. In rural area, during different seasons, food availability and affordability varies more than in urban area which may increase the within-person variations. Therefore, a larger sample size or a higher number of 24DRs per person would have likely resulted in a better estimation of correlations. However, SFFQ is designed for ranking individuals and additional analysis of ranking showed high agreement on classifying individuals in the same categories by both methods and confirmed the relative validity of our SFFQ.

The correlation coefficient is commonly accepted for measuring the strength of association between new methods against the comparison method or gold standard. However, this could be misleading as the correlation coefficient is not a measure of agreement between the two methods. We used the Bland-Altman method to assess the limit of agreement and showed that the two methods were comparable. The small mean of difference indicated that SFFQ2 slightly underestimated energy, protein and fat in both urban and rural areas and over-estimated carbohydrate intake. Additional analysis of ranking showed high percentage of individuals in the same categories by both methods and confirmed the relative validity of our FFQ.

The reproducibility of the SFFQ was moderate, and for most nutrients the correlation coefficients between two SFFQs were approximately greater than 0.4. Cade et al. [5] suggested a threshold of 0.4 and Masson suggested threshold above 0.5 [26] as acceptable reproducibility. The reproducibility correlation may be higher among studies conducted in Western countries as people do not change their food habits in a short period of time. Nutrition transition which is happening in low and middle income countries, causes rapid dietary change and may explain the lower correlation in our study in comparison with some other studies [16]. Higher correlation coefficients might have been observed if the two SFFQs had been administered only a few months apart.

Few studies examined validation of FFQs in Latin and Central America [7], [11], [27]. We are aware of only one other study conducted in Cordoba -Argentina by Navarro et al. (2001) among individuals aged 23–80 y [11]. Participants of that study (n = 62) were control subjects of a case-control study. Similar to our study, Navarro et al. used four 24DRs as the comparison method. The observed correlation between FFQ and 24DRs varied from 0.51 to 0.74 and were stronger than estimated correlations in our study. This might happen because Navarro study administered four 24DRs only 20 days apart while we collected 24DRs in four different seasons. However, we were unable to use their FFQ as dietary habit varies substantially in different areas of Argentina. Also, Navarro study did not include people from rural areas.

The present study is the first study that has developed and validated a SFFQ in both urban and rural areas of Argentina. There is no single gold standard way of developing an FFQ or assessing its validity and reliability. However, the methods used in our study, including selection of population, sample size, standard process of SFFQ development, and statistical approaches, were consistent with commonly accepted practices. The standard methods of data collection in both urban and rural areas, using colored food photographs, and completion of 24DRs and SFFQs by the same interviewers make the SFFQ a valid tool for measuring individual habitual intake.

This study has some limitations. For FFQ validation, multiple 24DRs are mostly used as comparison method (75% of validation studies), although biomarkers are the gold standard for some nutrients [5]. However, this study is part of an ongoing cohort study and we used only four 24DRs in urban and three 24DRs in rural as comparison method. The estimate of intake may have been closer to true intake if we had collected more than three and four days of food consumption or used biomarkers as the gold standard. Both SFFQ and comparison methods rely on participants’ memory to a certain extent and are prone to similar measurement errors. Participant cooperation is a limiting factor when a study is conducted in rural area or less privileged populations, though, the participants of this study were a highly motivated sample of the rural setting which might reduce the measurement errors. We informed participants of the day of dietary assessments, hence participants may have changed their diet on those days. We excluded 14 participants from the present analyses. Although their demographic characteristics were similar to other participants, loss of follow up may slightly bias the observed correlations.

In conclusion, this 96-item food frequency questionnaire had moderate relative validity and can be used to rank individuals based on macro and micro-nutrient intakes. The relative validity of SFFQ indicated that important associations between diet and disease can be measured for most nutrients; however, for a few nutrients with high within-person variation (such as fat intake), the precision of measurement may increase by stratified analysis for urban and rural settings. The validation study was conducted in unique settings with food cultures of both urban and rural areas included, which increased the applicability of the outcome when employed to a larger study such as PURE.
